# Putamen Atrophy as a Predictive Factor of Efficacy of GPi‐DBS in Dystonia‐Dyskinesia Syndrome Secondary to Perinatal Anoxic Encephalopathy

**DOI:** 10.1002/mds.70275

**Published:** 2026-03-18

**Authors:** Marylou Grasso, Pierre‐Olivier Moser, Sidonie Sauvageot, Valérie Gil, Emilie Chan‐Seng, Emily Sanrey, Philippe Coubes, Gaëtan Poulen

**Affiliations:** ^1^ Research Unit (URCMA: Unité de Recherche sur les Comportements et Mouvements Anormaux), Centre Hospitalo‐Universitaire Gui de Chauliac Montpellier France; ^2^ Stereotactic and Functional Neurosurgery Unit, Département de neurochirurgie, Unité «Pathologie Cérébrales Résistantes», Centre Hospitalo‐Universitaire Gui de Chauliac Montpellier France; ^3^ MMDN, University of Montpellier, EPHE, INSERM Montpellier France

**Keywords:** Cerebral Palsy, Deep Brain Stimulation, Dyskinesia, Dystonia, Globus Pallidus Intern, Pallidum, Perinatal Anoxic Encephalopathy, Putamen

## Abstract

**Background:**

Perinatal hypoxic–ischemic encephalopathy (HIE) is a severe condition resulting from impaired oxygen delivery to the developing brain, often leading to both motor deficits and dystonia‐dyskinetic syndromes (DDS). In selected cases, deep brain stimulation of the globus pallidus internus (GPi‐DBS) may provide a therapeutic option. However, predicting outcomes remains challenging because of clinical heterogeneity and variable responses.

**Objectives:**

This retrospective study aims to identify preoperative imaging predictors of GPi‐DBS efficacy in patients with DDS secondary to HIE, focusing on putaminal atrophy as a potential criterion.

**Methods:**

We retrospectively analyzed 73 patients with DDS secondary to HIE who underwent GPi‐DBS at our institution from 2003 to 2023. Clinical outcomes were assessed using the Burke‐Fahn‐Marsden Dystonia Rating Scale (BFMDRS) and Barry‐Albright Dystonia Scale (BADS) at baseline and up to 15 years post‐surgery. Preoperative magnetic resonance imaging scans were qualitatively and quantitatively evaluated to assess putaminal atrophy. Statistical analyses explored the relationships between imaging findings, clinical severity, and DBS outcomes.

**Results:**

Patients with severe putaminal atrophy exhibited significantly higher preoperative BFMDRS motor and disability scores, correlating with a limited response to DBS at 1‐year follow‐up (*P* < 0.05). Volumetric analysis confirmed that greater putaminal atrophy was associated with poorer motor improvements post‐surgery. The predictive value of putaminal volume for long‐term outcomes remained significant at 5‐year follow‐up.

**Conclusions:**

Putaminal atrophy is a key predictor of suboptimal outcomes following GPi‐DBS in patients with HIE‐related DDS. These findings highlight the importance of preoperative imaging in candidate selection and underscore the need for alternative strategies in patients with severe post‐anoxic basal ganglia damage. © 2026 The Author(s). *Movement Disorders* published by Wiley Periodicals LLC on behalf of International Parkinson and Movement Disorder Society.

Perinatal hypoxic–ischemic encephalopathy (HIE) is a severe neurological condition resulting from impaired oxygen delivery to the child's brain. It can arise from various causes, including prenatal hypoxia and complications during delivery with perinatal asphyxia. The incidence of HIE ranges from 1.5 to 8 per 1000 live births in developed countries and is higher in low‐resource settings because of inadequate access to prenatal and perinatal care.^1^ HIE is a major cause of neonatal mortality and long‐term neurodevelopmental disabilities, posing significant management challenges.

Clinically, HIE presents with a broad spectrum of neurological symptoms, depending on the severity of hypoxic–ischemic injury. Motor symptoms vary widely, depending on the extent of post‐anoxic lesions.^2^, ^3^ Dystonia‐dyskinesia syndrome (DDS) is a prominent but rarely isolated feature in HIE, characterized by sustained or intermittent muscle contractions that cause twisting movements, repetitive motions, or abnormal postures.^4^

The prevalence of DDS in HIE is high.^5^, ^6^ Management requires a multidisciplinary approach. Specific magnetic resonance imaging (MRI) findings associated with HIE include thalamic and basal ganglia lesions, white matter damage, and/or cortical atrophy, particularly in central regions.^7^

Basal ganglia lesions are now established as a key prognostic marker in HIE. MRI abnormalities in these regions strongly predict long‐term motor and cognitive outcomes. In a large cohort of 175 neonates, Martinez‐Biarge et al^8^ demonstrated a strong correlation between the severity of basal ganglia‐thalamus lesions on neonatal MRI and long term‐motor impairment (*r* = 0.77; *P* < 0.001). Severe lesions predicted severe cerebral palsy (Growth Motor Function Classification System GMFCS IV/V) with 96% sensitivity, 77% specificity.^8^ These findings highlight the central prognostic role of basal ganglia integrity in HIE.

Several MRI scoring systems have been proposed to grade neonatal hypoxic–ischemic lesions.^9^, ^10^, ^11^, ^12^ Across all systems, basal ganglia injury consistently emerges as the strongest predictor of adverse motor and developmental prognosis. However, these scoring systems do not account for the potential impact of DBS treatment or its possible efficacy.

The efficacy of pharmacological treatments is often limited, and side effects can be significant, particularly in pediatric populations.^13^

Deep brain stimulation (DBS) of the motor globus pallidus internus (GPi) has been successfully used in children with genetically determined DDS, such as TOR1A variants (formerly DYT1), myoclonus‐dystonia because of pathogenic SGCE variants (formerly DYT11), and KMT2B pathogenic variants,^14^, ^15^, ^16^ and was subsequently explored for movement disorders combining pyramidal and extrapyramidal features.^15^, ^17^, ^18^, ^19^

Several studies have investigated GPi‐DBS in pediatric populations, particularly for dystonia secondary to HIE.^3^, ^20^, ^21^ Although initial results suggest potential improvements in motor function and quality of life,^19^ outcomes across studies remain heterogeneous, the existing literature does not yet support definitive conclusions, and the mechanism of action of DBS remains incompletely understood.

Given the central role of cortico‐striatal pathways in these circuits, particular attention has been directed toward the motor putamen. In early brain injury such as HIE, putamen is particularly vulnerable, because it represents the main entry point for cortical motor inputs into the striato‐pallidal circuits. This perspective is consistent with broader evidence across movement disorders, demonstrating that both structural and functional connectivity strongly predict DBS outcomes. In cervical dystonia, pallido‐putaminal connectivity explains up to 68% of the variance in clinical outcome.^22^ In essential tremor, therapeutic success depends on stimulation site connectivity with the dentato‐rubro‐thalamic tract.^23^ More broadly, both functional and structural connectivity profiles have been shown to predict initial DBS responses^24^, ^25^ reinforcing the concept that DBS efficacy depends on modulation of specific neural circuits rather than isolated anatomical regions.^26^

Available literature suggests that DBS may benefit carefully selected patients with severe, medication‐refractory dystonia.^27^ Koy et al^28^ demonstrated an average improvement of DIS (Dyskinesia Impairment Scale) total scores after pallidal DBS in 14 pediatric patients with HIE over 36 months, however, individual patient responses were heterogeneous and some patients showed limited or no benefit. No significant changes were observed in other outcome measures, and during the 36‐month follow‐up, 12 serious adverse events in nine patients and 19 treatment‐related or possibly treatment‐related adverse events were reported. The authors concluded that larger, more homogeneous studies are necessary to clarify DBS's role in treating HIE.

Our study retrospectively included all patients who underwent GPi‐DBS for DDS post HIE in our center, and we evaluated both the motor response to stimulation and potential imaging‐based predictors of outcome.

## Patients and Methods

### Study Population

We conducted a retrospective analysis of patients from our prospective database. Inclusion criteria were age 6 or more years and GPi‐DBS treatment between 2003 and 2023 for DDS secondary to HIE. All patients were treated by the functional and stereotactic neurosurgery team at Montpellier Hospital.

HIE diagnosis was typically established at birth based on clinical evidence, with early manifestations such as tone abnormalities, delayed motor development, and abnormal movements. Perinatal anoxia was confirmed through detailed medical history, including pregnancy records, delivery complications, and neonatal course.

Exclusion criteria included genetic forms of generalized dystonia and DDS caused by alternative etiologies, such as infection, trauma (postnatal traumatic brain injury), toxicity, or hemorrhage.

### Clinical Assessment

Each patient underwent a preoperative evaluation by at least one neurologist and one neurosurgeon with expertise in movement disorders. These evaluations were supplemented by retrospective clinical assessments using standardized video recordings before surgery, at 1 year, at 5 years, and every 5 years thereafter until the last follow‐up.

The Burke‐Fahn‐Marsden Dystonia Rating Scale (BFMDRS) was used to assess dystonia severity (more data are available in Data S1).

### MR Imaging Acquisition

All imaging was performed on 1.5 T MRI scanners (Siemens Medical Solutions, Erlangen, Germany). The MRI protocol included standardized sequences such as T1‐weighted, T2 turbo spin echo‐weighted, susceptibility weighted imaging, and fluid‐attenuated inversion recovery images, with slice thicknesses ranging from 1 to 2 mm.

### MR Imaging Qualitative Analysis

Preoperative brain MRI images were retrospectively collected from patient records and analyzed using Myrian software. Two observers visually inspected the MRI images to identify the location and type of lesions. Atrophy, assessed on T1‐weighted images, and hyperintensities, assessed on T2‐weighted images, was noted in several brain regions, especially the basal ganglia and thalamus.

To refine this classification, we conducted quantitative volumetric measurements using manual and automated segmentation methods (more data are available in Data S1). To assess the accuracy of our morphological distribution groups, we compared the volumes obtained using the two quantitative methods within each group.

### Putamen MRI‐Volumetric Measurement

Volumetric segmentation of the putamen was performed using both manual and automated methods on T1‐weighted images. Manual segmentation was carried out using Horos software (v 3.3.6, USA), an open‐source medical image viewer based on OsiriX. This process involved tracing the boundaries of the putamen slice by slice, after which the resulting regions of interest (ROIs) were combined to calculate the total volumes for the left and right putamen independently. For these analyses, only the residual putaminal tissue was measured, with atrophic extensions displaying a “radish‐tail” appearance systematically excluded. Conversely, T2 hyperintensities within the putamen were included in the volumetric measurements, because they represent intrinsic pathological alterations of the structure. Automated segmentation was performed using BrainLab software (Boston Scientific). All volumes were expressed in cubic centimeters (cm^3^).

To validate the manual volumetric measurements of the putamen, a reproducibility study was conducted on 10 patients. For intra‐observer reproducibility, the same experimenter repeated the manual segmentation on 10 randomly selected patients. For inter‐observer reproducibility, a second blinded evaluator independently performed the segmentation using the same method.

### Gray Matter MRI‐Volumetric Measurement

Automated volumetric analysis of gray matter was performed using the CAT 12 toolbox implemented in SPM. Quality control was systematically applied, and volumes were visually inspected to ensure the accuracy of segmentation.

### Statistical Analysis

Statistical analysis was conducted using GraphPad Prism software (version 10.3.1). To compare preoperative BFMDRS motor and disability scores and Barry‐Albright Dystonia Scale (BADS) scores with postoperative scores at 1, 5, 10, and 15 years, paired *t* tests were used. Putaminal volumes were compared between groups using unpaired *t*‐tests, while thalamic volumes were compared between patients with and without atrophy using the same test. Gray matter volumes were analyzed with the Mann–Whitney test for diffuse atrophy and with unpaired *t* tests for central atrophy as well as for the putaminal and thalamic atrophy groups.

The association between the presence or absence of thalamic atrophy and both preoperative severity and postoperative outcomes were assessed using the Mann–Whitney test. Correlations between putamen volume or thalamic volume and clinical severity or postoperative improvement at various time points were assessed using Pearson correlation coefficients. Reproducibility of volumetric measurements was evaluated using paired *t* tests, while comparisons between manual and automated volumetric methods were made using unpaired *t*‐tests. Backward stepwise multivariate regression was performed to adjust for potential confounders (age at MRI, sex, and preoperative severity), with postoperative motor improvement (%) as the dependent variable and putaminal volume as the independent variable of interest.

Finally, post hoc analyses were conducted using receiver operating characteristic (ROC) curves to evaluate BFMDRS motor, BFMDRS functional, and BADS variables between the two atrophy groups. Threshold values for each variable were determined, along with associated sensitivity and specificity, and high sensitivity was prioritized to enhance case identification. Additionally, the area under the curve (AUC) and likelihood ratio indices were calculated to further assess diagnostic performance.

## Results

### Study Population

Seventy‐three patients met the inclusion criteria of our cohort. The mean age at surgery was 23 years (range, 6–65), with a sex ratio of 1.2 (male/female, 40/33). All patients exhibited a generalized DDS resulting from HIE, which had persisted since childhood, and for whom GPi‐DBS was anticipated. Regarding perinatal data, 67 pregnancies (91.8%) were carried to term. Two newborns (1.5%) were premature, and four (5.5%) were post‐term. At birth, 27 newborns (75%) were in a “state of apparent death,” 47 (88.7%) required resuscitation, and 17 had neonatal epileptic seizures. Eight newborns experienced all three conditions. Admission to intensive care unit was necessary for 29 patients (69%), whereas 21 patients (58.3%) required assisted mechanical ventilation, with 13 (7.8%) experiencing all three conditions.

Thirty patients underwent surgery before the age of 18, and 43 were operated on after 18 years old. Seventy‐one patients were implanted with Medtronic leads (3389), and recently, two patients received Boston Scientific Cartesia leads. Nine patients were implanted with a rechargeable IPG, while 64 patients received a non‐rechargeable IPG (Internal Pulse Generator). Ten patients initially implanted with a non‐rechargeable IPG later received a rechargeable IPG. According to our protocol, the placement of the IPG was in the abdominal area for 66 patients and in the sub mammary area for seven female patients.

Postoperative MRI systematically confirmed accurate electrode placement within the sensorimotor portion of the GPi (Fig. S1). No immediate postoperative complications were reported, including any intracerebral hematomas (as confirmed by immediate stereotactic magnetic resonance [MR] control and a day 5 computed tomography [CT] scan). Two patients experienced delayed local infections, leading to DBS system removal at 2 and 7 years postoperatively.

### Clinical Outcome

The mean preoperative BFMDRS motor score was 58.5 (range, 16.5–104), while the mean preoperative BFMDRS disability score was 16.9 (range, 2–30). The mean preoperative BADS score was 18.6 (range, 9–26). One year after surgery, a statistically significant improvement was observed, with the average BFMDRS motor and disability scores decreasing to 48.8 (range, 0–96; *P* < 0.0001) and 15.9 (range, 0–30; *P* < 0.0001), respectively. The BADS score also improved to 17.6 (range, 0–26; *P* < 0.0001).

These improvements remained stable over time. At 5 years post‐DBS (n = 55), the results were consistent. At 10 years post‐DBS (n = 17) and 15 years post‐DBS (n = 14), significant improvements in the BFMDRS motor score persisted (*P* = 0.0020 and *P* = 0.0148, respectively). Additionally, the BFMDRS disability score remained improved at 15 years (*P* = 0.0322), although the BADS score showed no statistically significant long‐term changes.

Comparative analyses of BFMDRS movement, disability, and BADS scores across putamen atrophy groups revealed distinct trends. In group 1 (mild or no atrophy), the average preoperative BFMDRS movement and disability scores were 45.5 ± 16 (range, 16.5–88.75) and 11.8 (range, 2–27), with a BADS score of 16.4 (range, 9–24). Significant improvements were observed at 1 year in BFMDRS movement (*P* < 0.0001), disability (*P* < 0.0001), and BADS scores (*P* = 0.0013), and these gains were maintained at 5 years (*P* < 0.0001; *P* = 0.0005; *P* = 0.0333).

In group 2 (severe atrophy), the average preoperative BFMDRS movement and disability scores were 70.7 ± 19.9 (range, 20–104) and 22.2 ± 5.6 (range, 5–30), with a BADS score of 21.4 ± 3 (range, 14–26). Similar improvements at 1 year were observed in BFMDRS movement (*P* < 0.0001), disability (*P* = 0.004), and BADS scores (*P* = 0.0004). These improvements remained significant at 5 years for the BFMDRS motor and disability scales (*P* = 0.018 and *P* = 0.0283, respectively), but not for the BADS scale (*P* = 0.144). All data are summarized in Table 1 (more data are available in Data S1).

**TABLE 1:** . Patient characteristics, BFMDRS outcomes, and putamen volume correlation after 1, 5, 10, and 15 years of GPi DBS

Perinatal post‐hypoxic encephalopathy	Sample size	Sex ratio (M/F)	Mean age at surgery (yr)	Preoperative BFMDRS movement score	1 yr postoperative movement score mean (SD) (n = XX)	5 yr postoperative movement score mean (SD) (n = XX)	10 yr postoperative movement score mean (SD) (n = XX)	15 yr postoperative movement score mean (SD) (n = XX)
Total cohort	n = 73	40/33	23.5 (range: 7–65)	58.5 (± 23), n = 70	48.8 (±23.8), n = 63, *P* < 0.0001	47.2 (± 24.9), n= 55, *P* < 0.0001	46 (±27.2), n = 17, *P* = 0.0020	54.7 (± 21.3), n = 14, *P* = 0.0148
Group 1: no/mild motor putamen atrophy hypersignals	n = 31	23/8	27.1 (range: 9–65)	45.5 (±16.5), n = 31	34.7 (±18.4), n = 28, *P* < 0.0001	33.38 (±19.5), n = 27, *P* < 0.0001	NA	NA
Group 2: severe motor putamen atrophy and hypersignals	n = 33	16/17	21 (range: 7–63)	70.7 (± 19.9), n = 30	66.3 (±16.7), n = 28, *P* < 0.0001	63.10 (±16.74), n = 23, *P* < 0.0001	NA	NA

Abbreviations: BFMDRS, Burke‐Fahn‐Marsden Dystonia Rating Scale; GPi, globus pallidus internus; DBS, deep brain stimulation; M, male; F, female; SD, standard deviation.

### MRI Lesions

Sixty‐four preoperative MRIs were analyzed for lesion identification. The majority of patients exhibited putaminal abnormalities, with 58 patients (90.6%) showing putamen atrophy and 54 patients (84.4%) displaying hyperintensities. Among these cases, 31 exhibited no or mild atrophy with hyperintensities, whereas 33 presented severe atrophy with hyperintensities.

**FIG. 1:**
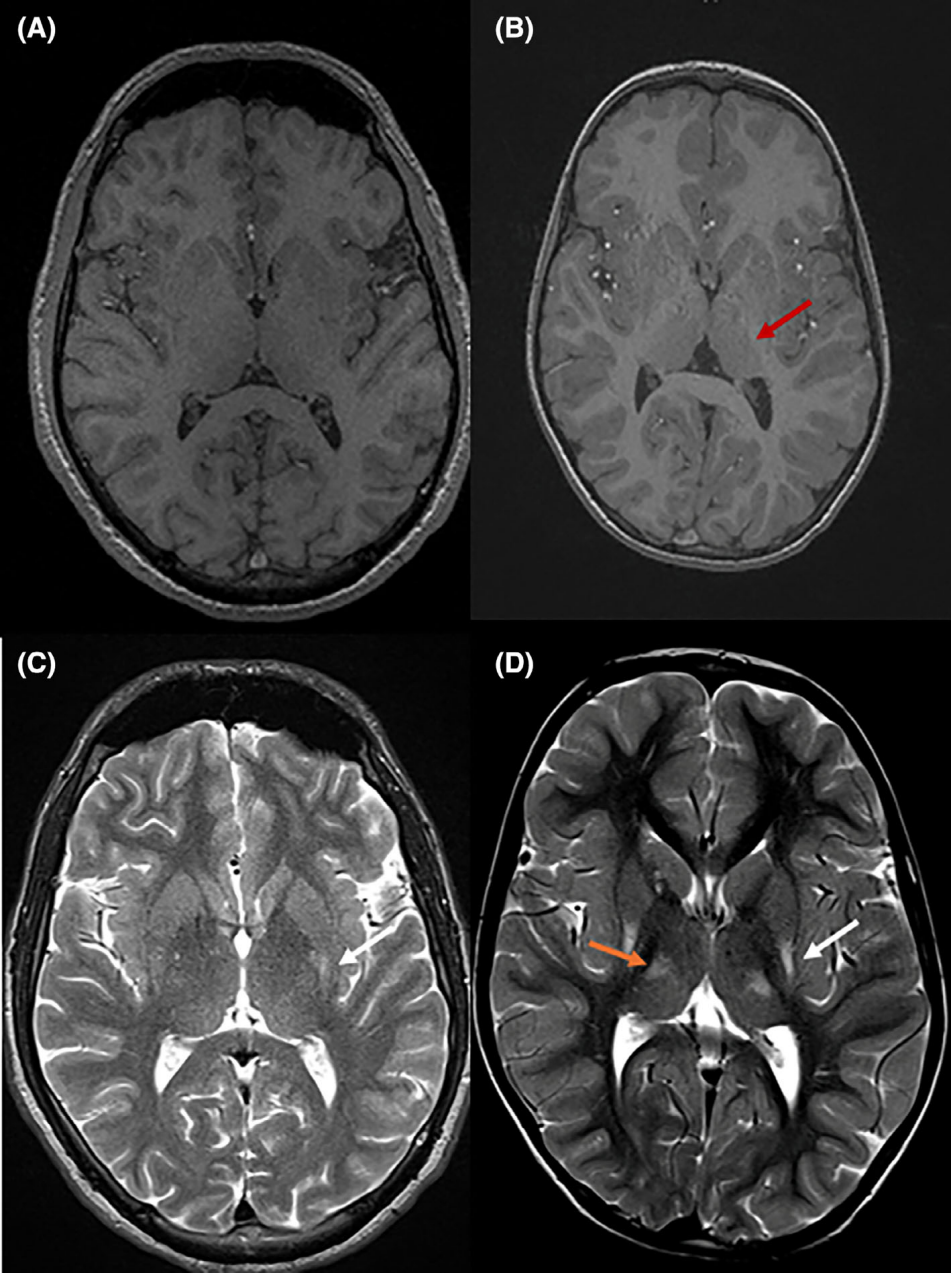
T1‐weighted (**A**) and T2‐weighted (**C**) magnetic resonance imaging (MRI) of the severity group 1 (no or mild putamen atrophy with hyperintensities) and T1‐weighted (**B**) and T2‐weighted (**D**) imaging of the severity group 2 (severe putamen atrophy and hyperintensities). The hyperintensities of the motor putamen are shown by the white arrows. In (**B**) and (**D**), thalamic atrophy (red arrow) and hyperintensities (orange arrow) with an “epsilon” appearance are also observed. [Color figure can be viewed at wileyonlinelibrary.com]

Lesions in the motor thalamus (Ventral intermediate nucleus/Ventro oralis posterior nucleus: Vim/Vop complex) were also observed, with 20 patients (35.9%) exhibiting atrophy and 44 patients (68.8%) showing hyperintensities (Fig. 1). These lesions were consistently bilateral. Additionally, diffuse cortical cerebral atrophy was present in 47 patients (73.4%), whereas hyperintensities in the white matter were noted in 17 patients (26.6%).

### Putamen Volumetry and Its Relationships with Neurological Severity and Clinical Outcome

Of the 64 MRI scans available for qualitative assessments, volumetric analysis was feasible in 54 cases because of image quality and availability of three dimensional T1 sequences with a sufficient number of slices.

The mean putamen volume in group 1 was 2.8 cm^3^ on the left side and 3.1 cm^3^ on the right side, whereas in group 2, it was 1.6 cm^3^ on the left side (*P* < 0.0001) and 1.8 cm^3^ on the right side (*P* < 0.0001) based on manual volumetry (Fig. S2). A significant difference was also observed between the two groups using automated volumetry (Fig. S2).

**FIG. 2:**
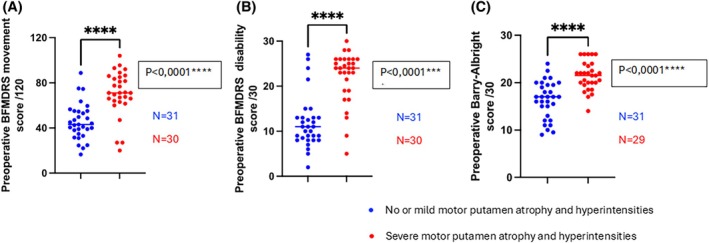
Comparison of motor scores between the “no or mild motor putamen atrophy and hyperintensities” group and the “severe motor putamen atrophy with hyperintensities” group using the preoperative Burke‐Fahn‐Marsden Dystonia Rating Scale (BFMDRS) movement score (A), the BFMDRS disability score (B) and the Barry‐Albright score (C). [Color figure can be viewed at wileyonlinelibrary.com]

**FIG. 3:**
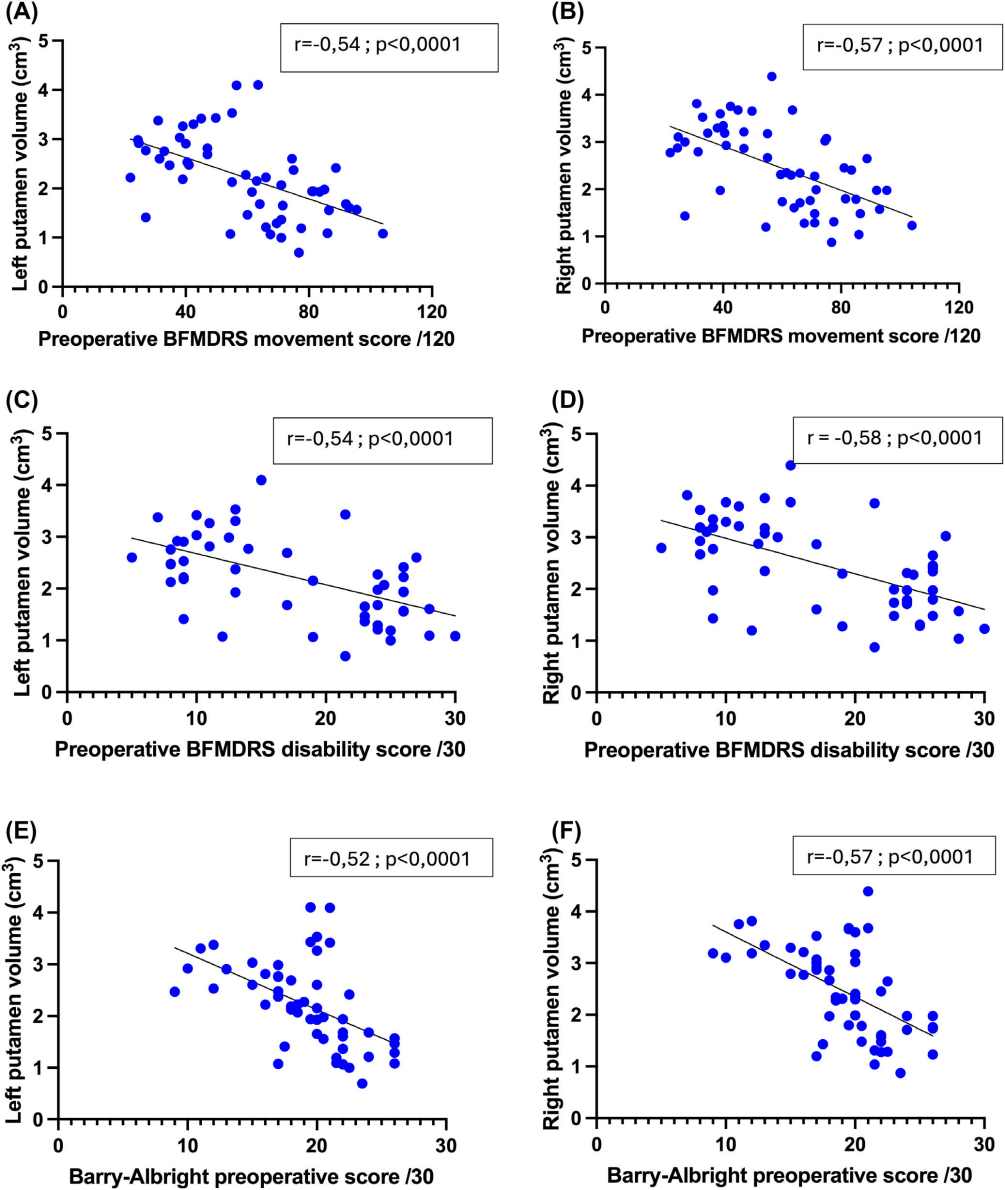
Correlations between the preoperative Burke‐Fahn‐Marsden Dystonia Rating Scale (BFMDRS) movement score and the left (**A**) and right (**B**) putamen volume. Correlations between the preoperative BFMDRS disability score and the left (**C**) and right (**D**) putamen volume. Correlations between the preoperative Barry‐Albright score and the left (**E**) and right (**F**) putamen volume. [Color figure can be viewed at wileyonlinelibrary.com]

**FIG. 4:**
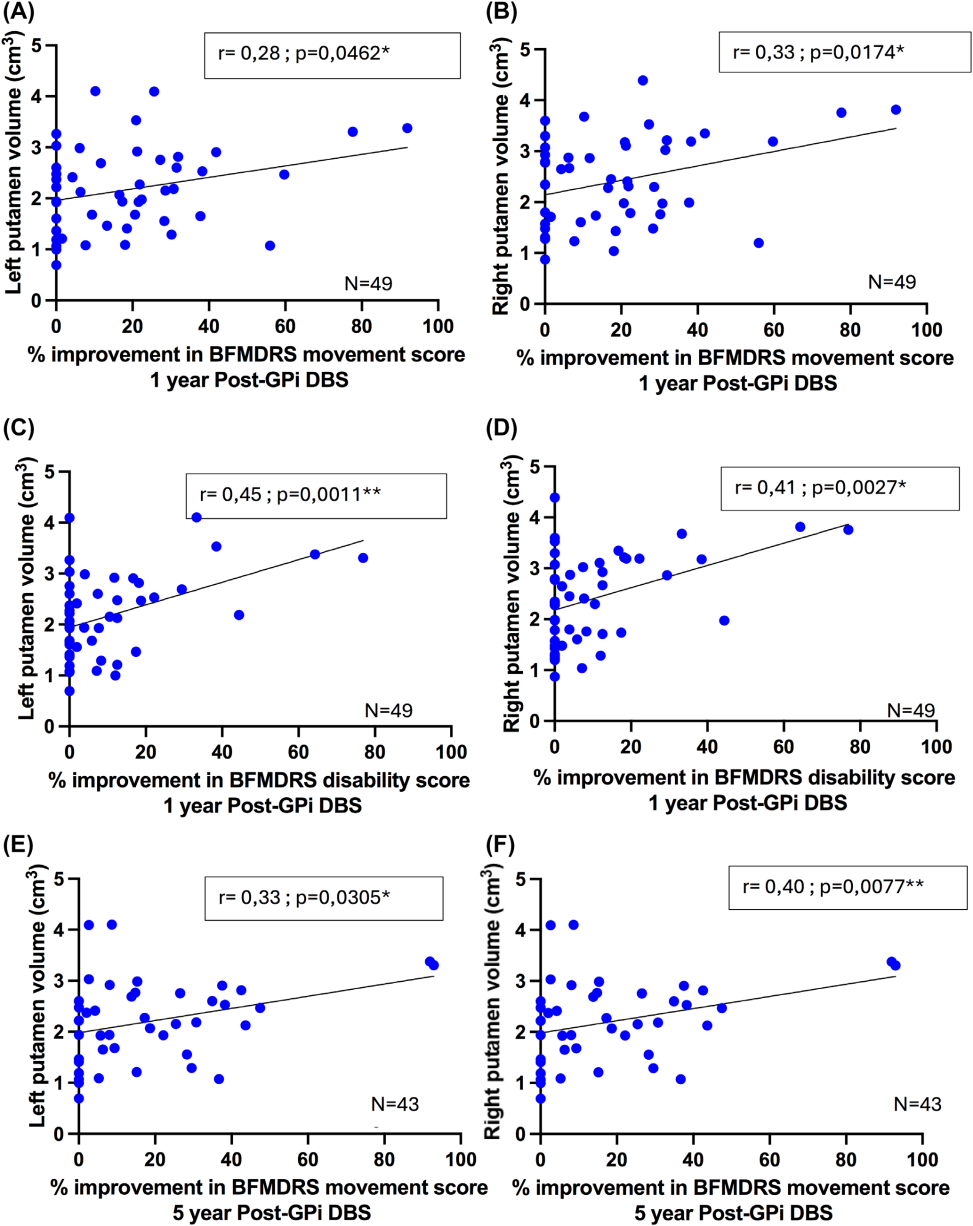
Correlation between the percentage of improvement on the 1‐year Burke‐Fahn‐Marsden Dystonia Rating Scale (BFMDRS) movement score and the left (**A**) and right (**B**) putamen volume. Correlation between the percentage of improvement on the 1‐year BFMDRS disability score and the left (**C**) and right (**D**) putamen volume. Correlations between the 5‐year BFMDRS movement score and the left (**E**) and right (**F**) putamen volume. [Color figure can be viewed at wileyonlinelibrary.com]

Patients in group 1 had significantly lower preoperative BFMDRS motor, BFMDRS disability, and BADS scores compared to group 2 (*P* < 0.0001 for all three scales) (Fig. 2). A correlation was observed between putamen volume (left or right) and clinical severity at preoperative assessment, as reflected in the BFMDRS motor, BFMDRS disability, and BADS scores (Fig. 3). Greater putaminal atrophy was associated with more severe preoperative motor impairment. Similarly, a linear correlation was identified between putamen volume and response to GPi‐DBS at 1‐year follow‐up for BFMDRS motor and disability scores: *r* = 0.28 and *P* = 0.0462 for BFMDRS motor and left putamen, *r* = 0.33 and *P* = 0.0174 for BFMDRS motor and right putamen, *r* = 0.45 and *P* = 0.0011 for BFMDRS disability and left putamen, and *r* = 0.41 and *P* = 0.0027 for BFMDRS disability and right putamen (Fig. 4).

Smaller putamen volume, indicating greater atrophy, was associated with poorer clinical outcomes. This correlation persisted at the 5‐year assessment between left and right putamen volume and the percentage of improvement on BFMDRS motor score (left putamen: *r* = 0.33, *P* = 0.0305; right putamen: *r* = 0.40, *P* = 0.0077) (more data are available in Data S1). No significant correlations were found at 10 and 15 years of follow‐up, likely because of the smaller number of patients.

### Impact of Global Gray Matter Volume

To account for potential confounding by global gray matter volume (GMV), total GMV were first compared between patients with and without diffuse atrophy, showing significantly lower values in the atrophy group (*P* = 0.0263), whereas no difference was observed when considering central atrophy (*P* = 0.0939). To further exclude the possibility that the relationship between motor putaminal atrophy and preoperative severity and postoperative outcomes merely reflected global gray matter loss, and GMV was compared between the two groups of putamen atrophy, yielding no significant difference (*P* = 0.6045). Similarly, difference in global GMV was found between patients with and without motor thalamic atrophy (*P* = 0.4446).

### Reproducibility of Putamen Measurement

To assess measurement reliability, we conducted an intra‐observer reproducibility analysis by having the same evaluator repeat measurements on 10 randomly selected subjects. No significant differences were found between the two measurements. Similarly, an inter‐observer reproducibility analysis was performed, involving a second blinded evaluator who independently measured putamen volume in the same 10 patients using identical methods. Again, no significant differences were identified.

However, when comparing manual volumetric measurements obtained using Horos software with automated volumetric data from BrainLab software, significant differences emerged. Automated volumetry tended to overestimate putamen volume, likely because of algorithmic limitations that failed to account for severe atrophy. These findings underscore the importance of manual validation in MRI volumetric assessments. These results are summarized in Figure S4.

### Backward Multivariate Regression Models for Motor and Functional Outcomes

At 1 year postoperatively, multivariate analyses revealed that putaminal volume is the only independent predictor of clinical improvement. Larger left and right volume were significantly associated with greater BFMDRS motor improvement (β = 7.26, 95% CI [0.13–14.3], *P* = 0.046; β = 8.08, 95% CI [1.49–14.66], *P* = 0.017; *R* ^2^ ≈ 0.14). A similar pattern was observed for functional outcomes, with both left and right putaminal volumes emerging as robust predictors of BFMDRS functional improvement (β = 9.17, 95% CI [3.85–14.50], *P* = 0.001; β = 8.02, 95% CI [2.92–13.12], *P* = 0.003; *R* ^2^ = 0.18–0.20). At 5 years, the predictive role of putaminal volume persisted for BFMDRS motor improvement (left: β = 9.13, 95% CI [0.90–17.36], *P* = 0.031; right: β = 10.35, 95% CI [2.89–17.82], *P* = 0.008). Age at the MRI acquisition, sex, and preoperative severity consistently failed to reach significance in any of the models.

### Post‐hoc Analysis

Preoperative analyses identified threshold values predictive of severe putaminal atrophy, rather than DBS outcome per se. For a BFMDRS motor score threshold of 59.7, sensitivity was 84.6% and specificity was 88.8%. For a BFMDRS functional score threshold of 15.5, sensitivity reached 92.5%, with a specificity of 84.6%. A BADS score threshold of 19.75 yielded a sensitivity of 77.8% and specificity of 76.9%. The AUC values were 0.85, 0.88, and 0.87 for the respective scales (Fig. S5). Likelihood ratios of 5.77 for the BFMDRS motor scale, 6.01 for the functional scale, and 3.3 for the BADS scale support these threshold values as predictive markers for GPi‐DBS response.

## Discussion

In the modern era, the development of medical possibilities for diagnosis and imaging in neurology has allowed better delineation of post‐natal neurological handicaps. Advances in MR imaging and neurogenetics have largely replaced the systematic use of the “cerebral palsy” concept, enabling more precise categorization of perinatally diagnosed encephalopathies. Concurrently, genetic disorders that can clinically mimic cerebral palsy, such as KMT2B‐related dystonia, have been increasingly recognized and may show favorable responses to GPi‐DBS, underscoring the importance of contemporary etiological stratification in patients with early‐onset dystonia. In this study, we described the evolution of selected cases of movement disorders with evidence on MRI, of perinatal HIE, without identified genetic mutation, treated with GPi‐DBS.

DBS has been first evaluated for treating tremor and Parkinson's disease patients. Second, our team proposed stereotactic MRI‐guided implantation of electrodes in the motor GPi under general anesthesia in a subgroup of children with dystonia‐dyskinesia syndrome because of TOR1A and THAP1 variants, usually with normal MRI.^14^, ^15^, ^16^ Quite early, we had to manage patients with HIE presenting with dystonia and dyskinesia. The efficacy did not seem to be as significant as that observed in the primary group, and it took a long time to gain a better understanding of the pathophysiology of these neurological disorders. This has been often reported.^17^, ^19^, ^29^, ^30^ Nevertheless, the role of electrical continuous motor GPi neuromodulation in DDS secondary to anoxia remains controversial because of the heterogeneity of results and the challenges in identifying the optimal candidates.

Extensive brain damage is a common feature in DDS secondary to HIE, with significant involvement of the basal ganglia, which are central to motor control. Our observations suggest a strong association between lesions in motor thalamic nuclei (eg, Vim, Vop) and severe putaminal atrophy, which correlate with variable outcomes following DBS. Lesion network mapping data indicate that 95% of causative lesions converge on motor thalamic territories, with preferential connectivity to sensorimotor parcellations over associative or limbic regions (analysis of variance [ANOVA] *P* = 0.0002; post‐hoc *P* < 0.001), suggesting that putamino‐thalamic disconnection may constrain the efficacy of GPi‐DBS in secondary dystonia.^31^

Retrospective analysis further confirmed that structural damage to key motor regions, including the putamen, is a hallmark characteristic in these patients. These damages reduce the efficacy of DBS, as supported by findings from Cajigas et al,^30^ who demonstrated that basal ganglia damage negatively impacts the outcomes of DBS targeting the GPi, although they did not specifically evaluate lesion volume. From a network perspective, structural injury to the motor putamen, reported in 43% of cases, likely compromises downstream thalamic integration within the basal‐ganglia‐thalamo‐cortical loop, thereby limiting the capacity of GPi stimulation to restore coherent motor network dynamics.^31^

Studies have reported improvements in motor function and quality of life, but these results are inconsistent across cohorts. For example, Koy et al^28^ conducted a long‐term follow‐up study on pediatric patients with dyskinetic HIE and found that although dyskinesia significantly improved in most patients, other non‐motor outcomes, such as quality of life, did not show consistent changes. This discrepancy may arise from the limitations of existing evaluation tools, which were designed for primary dystonia and may not adequately capture improvements in complex motor disorders like DDS. Interestingly, even slight or statistically insignificant motor improvement can result in meaningful enhancements in quality of life for these patients. The variability in quality of life outcomes may reflect differences in how these measures are assessed in this population, further emphasizing the need for disease‐specific evaluation tools.^29^

In our cohort, two patients showed exceptional motor responses, with improvements of 91.9% and 92.9% at 1‐ and 5‐year follow‐up. Such near‐complete responses are unusual, but have been reported in the literature. Romito et al^32^ described one patient achieving 86.1% improvement after GPi‐DBS, whereas the average motor benefit in their series was closer to 49%.These findings suggest that, although rare, extreme outcomes may occur under specific conditions. In our experience, such exceptional responses likely reflect a predominance of reversible dystonic/dyskinetic features rather than fixed structural deficits. Favorable clinical factors—including younger age at surgery, shorter disease duration before intervention, absence of severe skeletal deformities, and rigorous postoperative follow‐up—may further contribute to maintaining these improvements over time. Such cases underscore that, while structural injury often predicts limited benefit, carefully selected patients may nonetheless achieve remarkable and durable clinical responses.

Identifying preoperative factors of DBS efficacy is crucial for improving patient selection and optimizing outcomes. Our study's finding that putaminal atrophy predicts poor response to GPi‐DBS aligns with prior research emphasizing the role of structural integrity in DBS outcomes.^33^ Additional factors such as the age of the patient at surgery and the duration of symptoms have also been identified as important determinants of outcome.^34^ Early intervention, particularly before the development of severe musculoskeletal deformities or maladaptive neuroplasticity, appears to improve motor results.^35^

In conclusion, our study identifies putaminal atrophy as a critical predictor of poor outcomes following GPi‐DB in children with DDS secondary to HIE.

## Author Roles

(1) Research Project: A. Conception, B. Organization, C. Execution; (2) Statistical Analysis: A. Design, B. Execution, C. Review and Critique; (3) Manuscript Preparation: A. Writing of the First Draft, B. Review and Critique.

M.G.: 1B, 1C, 2B, 3A.

P.O.M.: 1C, 2B, 3B.

S.S.: 1C, 2B, 3B.

V.G.: 1C, 2B, 3B.

E.C.S.: 1C, 2B, 3B.

E.S.: 1C, 2B, 3B.

P.C.: 1A, 1B, 2C, 3B.

G.P.: 1A, 1B, 2C, 3B.

## Supporting information


**Figure S1.** Representative postoperative axial MRI showing bilateral electrode placement within the sensorimotor portion of the globus pallidus internus (GPi) (white arrow).

**Figure S2.** A: Left putamen manual volumetry. B: Right putamen manual volumetry. C: Left putamen automated volumetry. D: Right putamen automated volumetry.

**Figure S3.** Correlations between the preoperative BFMDRS disability score and the left (A) and right (B) thalamus volume. Correlations between the preoperative Barry‐Albright score and the left (C) and right (D) thalamus volume.

**Figure S4.** A: Assessment of the intra‐observer reproducibility. B: Assessment of the inter‐observer reproducibility. C: Comparison between manual (Horos) and automated (BrainLab) volumetry in the severity group 1 (no or mild putamen atrophy and hyperintensities). D: C: Comparison between manual (Horos) and automated (BrainLab) volumetry in the severity group 2 (severe putamen atrophy and hyperintensities).

**Figure S5.** Receiver operating characteristic (ROC) curves for the MRI classification model of motor putamen atrophy. The ROC curves illustrate the performance of our classification model in detecting motor putamen atrophy by comparing group 1 and group 2 patients. Three curves are displayed, corresponding to different clinical scale scores (BFMDRS movement and disability parts and BADRS). The area under the curve (AUC) for each score is indicated, reflecting the model's discriminative ability to differentiate between the groups.

**Data S1.** Supporting Information.

## Data Availability

The data that support the findings of this study are available on request from the corresponding author. The data are not publicly available due to privacy or ethical restrictions.
