# Supplementary figures and images for: Putamen Atrophy as a Predictive Factor of Efficacy of GPi‐DBS in Dystonia‐Dyskinesia Syndrome Secondary to Perinatal Anoxic Encephalopathy
Source: Mov Disord. 2026 Mar 18;41(6):1402–12. doi: 10.1002/mds.70275 (PMC13307266; doi:10.1002/mds.70275)

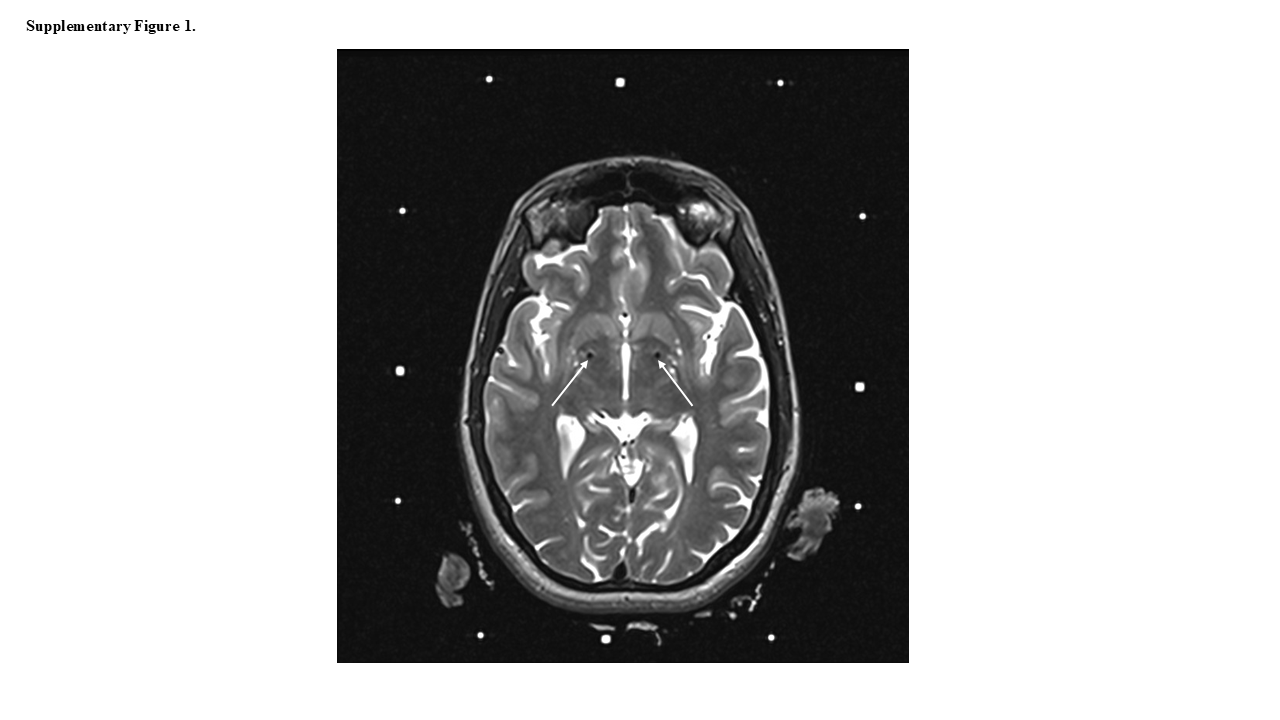

Supplement: Supplementary file 1 — Figure S1. Representative postoperative axial MRI showing bilateral electrode placement within the sensorimotor portion of the globus pallidus internus (GPi) (white arrow). [file MDS-41-1402-s003.tif]

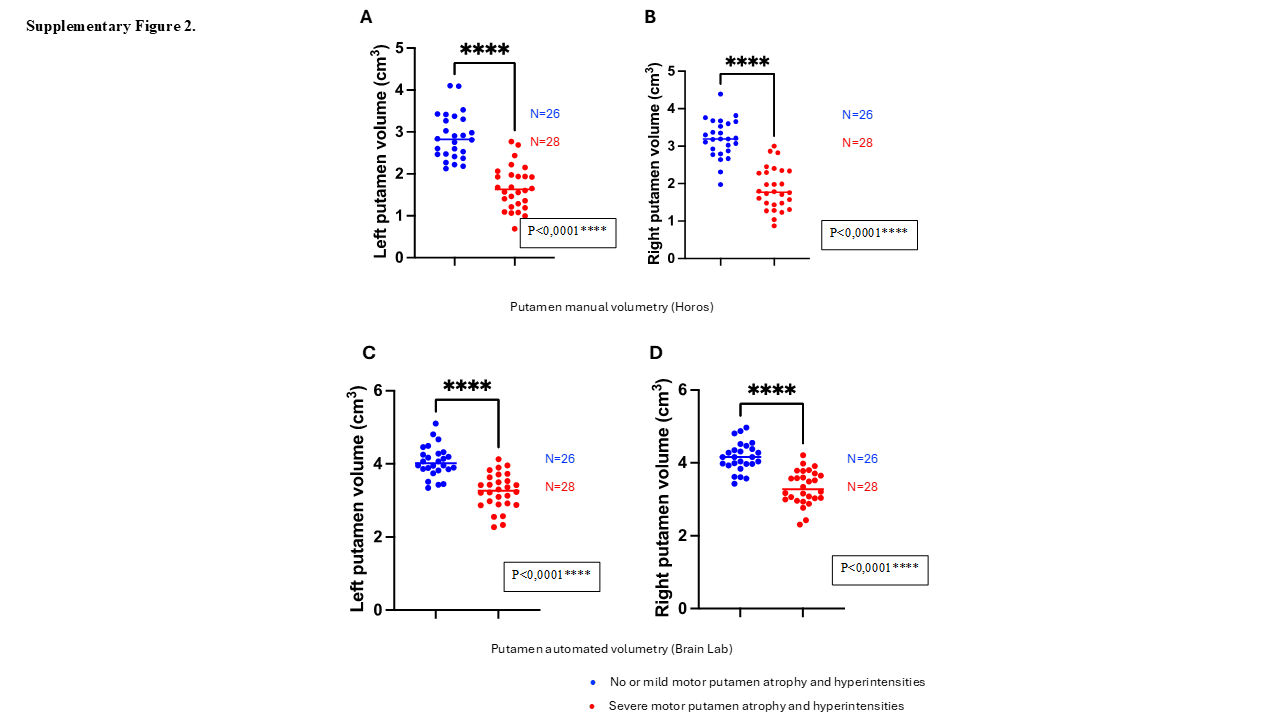

Supplement: Supplementary file 2 — Figure S2. A: Left putamen manual volumetry. B: Right putamen manual volumetry. C: Left putamen automated volumetry. D: Right putamen automated volumetry. [file MDS-41-1402-s006.tif]

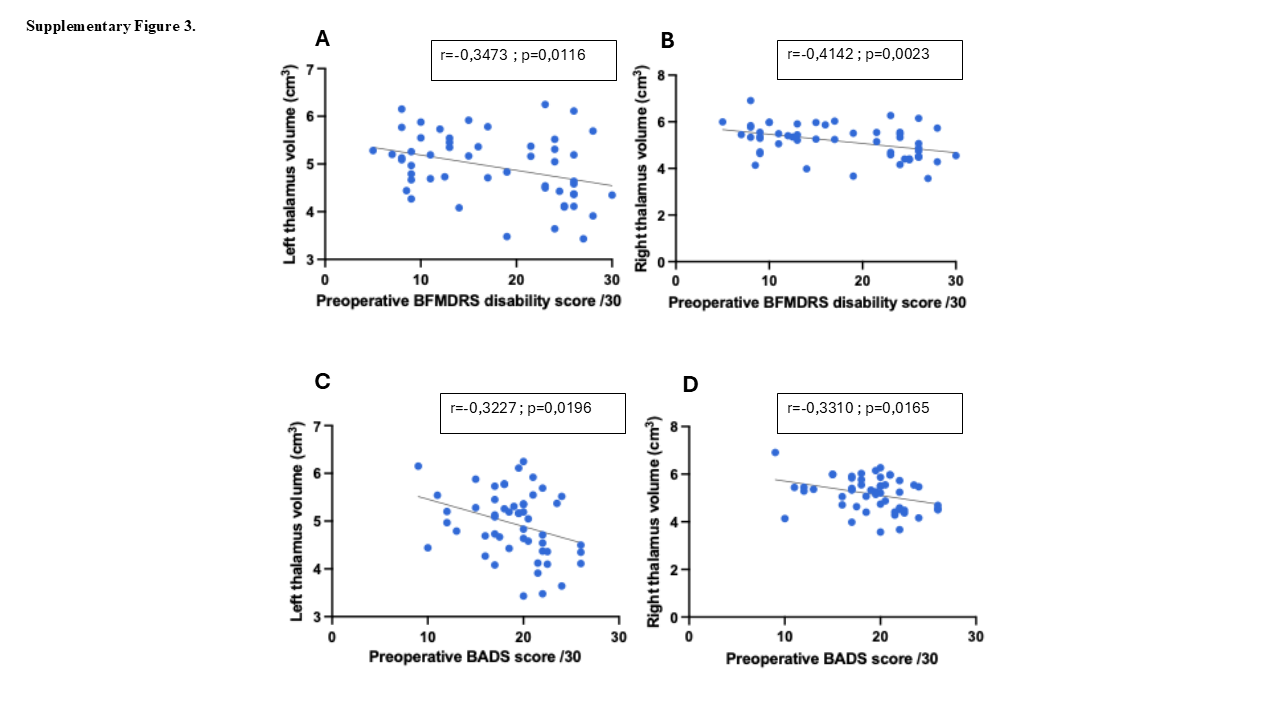

Supplement: Supplementary file 3 — Figure S3. Correlations between the preoperative BFMDRS disability score and the left (A) and right (B) thalamus volume. Correlations between the preoperative Barry‐Albright score and the left (C) and right (D) thalamus volume. [file MDS-41-1402-s005.tif]

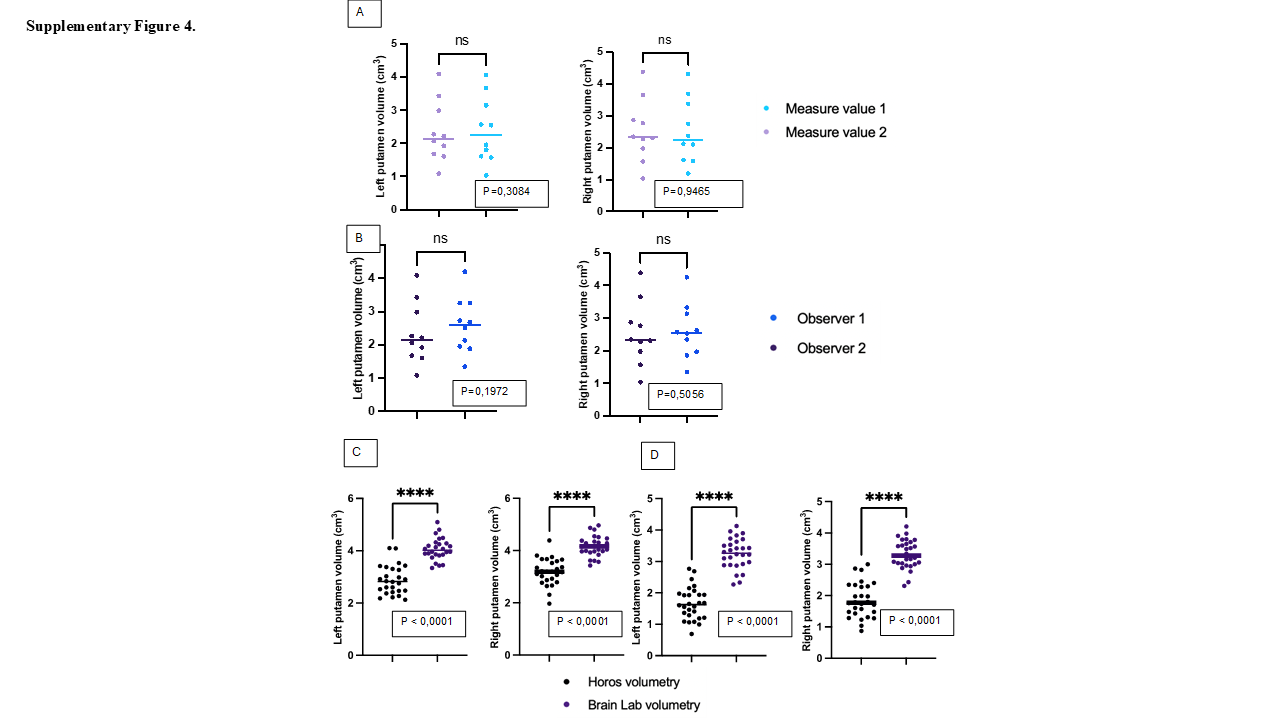

Supplement: Supplementary file 4 — Figure S4. A: Assessment of the intra‐observer reproducibility. B: Assessment of the inter‐observer reproducibility. C: Comparison between manual (Horos) and automated (BrainLab) volumetry in the severity group 1 (no or mild putamen atrophy and hyperintensities). D: C: Comparison between manual (Horos) and automated (BrainLab) volumetry in the severity group 2 (severe putamen atrophy and hyperintensities). [file MDS-41-1402-s001.tif]

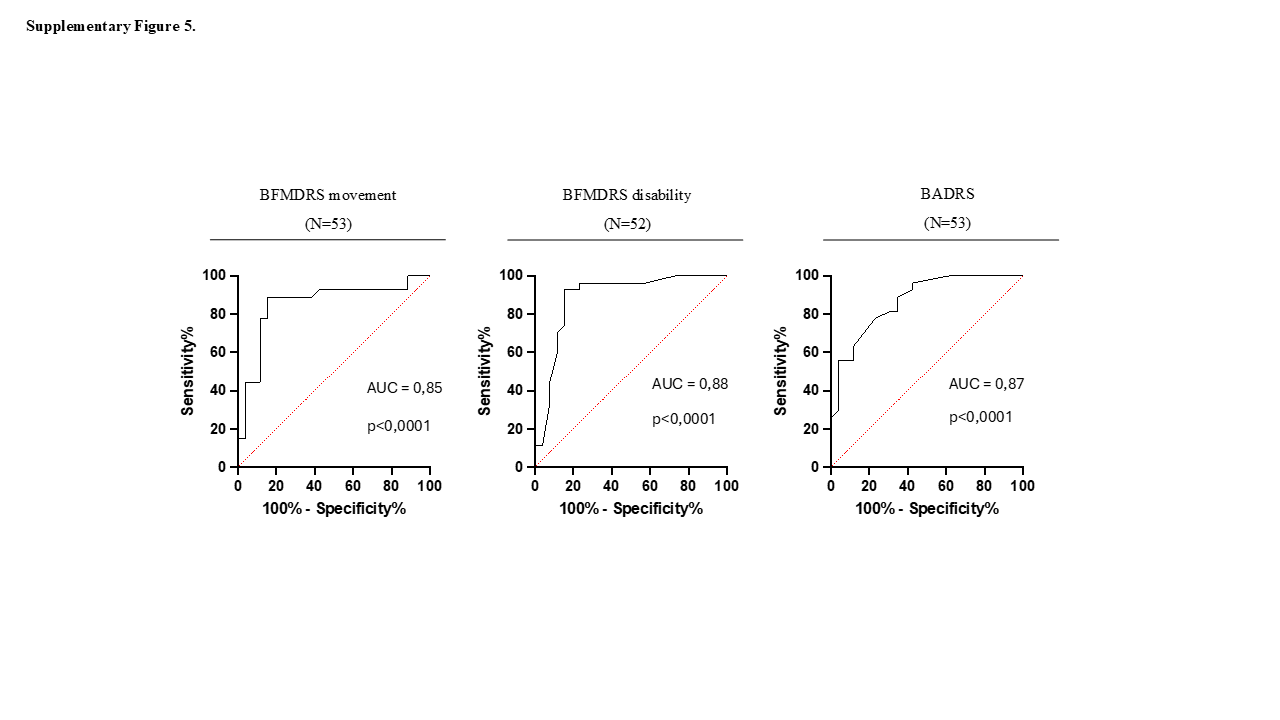

Supplement: Supplementary file 5 — Figure S5. Receiver operating characteristic (ROC) curves for the MRI classification model of motor putamen atrophy. The ROC curves illustrate the performance of our classification model in detecting motor putamen atrophy by comparing group 1 and group 2 patients. Three curves are displayed, corresponding to different clinical scale scores (BFMDRS movement and disability parts and BADRS). The area under the curve (AUC) for each score is indicated, reflecting the model's discriminative ability to differentiate between the groups. [file MDS-41-1402-s004.tif]
