# Supplementary material for: Putamen Atrophy as a Predictive Factor of Efficacy of GPi‐DBS in Dystonia‐Dyskinesia Syndrome Secondary to Perinatal Anoxic Encephalopathy
Source: Mov Disord. 2026 Mar 18;41(6):1402–12. doi: 10.1002/mds.70275 (PMC13307266; doi:10.1002/mds.70275)
Supplement: Supplementary file 6 — Data S1. Supporting Information. [file MDS-41-1402-s002.docx]

SUPPLEMENTARY DATA

SUPPLEMENTARY METHODS

**MRI-Guided Electrode Implantation Under General Anesthesia and programming**

DBS surgery followed our institutional protocol with MRI-based targeting. Postoperative programming was initiated with a frequency of 130 Hz, pulse width of 450 µs, and an initial voltage of 0.5 V, progressively increased based on clinical response and side effects. The programming phase lasted weeks to months to achieve optimal neuromodulation.

**Clinical assessment**

**The Burke-Fahn-Marsden Dystonia Rating Scale (BFMDRS) was used to assess dystonia severity. Two experienced evaluators independently performed the assessments, blinded to each other’s results and reached a consensus for each patient. In cases of minor discrepancies (≤10 points in the movement section and ≤5 points in the disability section), the average score was used. For larger discrepancies (>10 points in the movement section or >5 points in the disability section), video recordings were reviewed again to establish a final consensus. The Barry-Albright Dystonia Scale (BADS) was applied following the same methodology.**

**In our center a standardized video-based protocol enables the assessment of most disability items, including swallowing and hygiene assistance needs. These aspects are systematically addressed through structured or caregiver questioning during the video recording and cross-checked with information documented in the patients ’clinical records.**

**MR Imaging Qualitative Analysis**

**Regarding putaminal atrophy, we divided cases into two severity groups based on morphological analysis, without the use of a predefined volumetric cut-off: Group 1 included cases with no or mild atrophy, characterized by subtle posterior tapering with a “torch-like” with a “radish tail” appearance, with hyperintensities. Group 2 included severe atrophy, defined by marked volume loss and sclerosis, with hyperintensities (figure 1).**

For the thalamic analysis, patients were classified into two additional groups based on thalamic morphology on MRI, independently of putaminal involvement. Thalamic atrophy was defined by the presence of a characteristic “epsilon” sign involving the motor thalamic nuclei on T1-weighted images. Patients were accordingly categorized as with or without thalamic atrophy.

**Thalamic MRI-Volumetric Measurement**

Automated segmentation of the thalamus was performed on T1-weighted MRI images, using Brainlab software, which has been validated for subcortical structure delineation. The resulting thalamic volumes were visually inspected and manually corrected when necessary to account for potential artifacts or lesions. Final volumetric values were then extracted for quantitative analysis.

SUPPLEMENTARY RESULTS

**Thalamic volumetry and** **its relationships with neurological severity and clinical outcome**

Automated segmentation–based morphometric analysis revealed reduced thalamic volumes in patients with atrophy (left: 4.69 cm³; right: 4.98 cm³) compared with those without atrophy (left: 5.15 cm³; right: 5.33 cm³). The difference was statistically significant for the left thalamus (p = 0.0164), while the right side showed only a nonsignificant trend (p = 0.0874).

Thalamic involvement, either morphological (atrophy) or signal-related (hyperintensity), was associated with greater preoperative functional impairment, as reflected by higher BFMDRS-F score (p = 0.0468 and p = 0.0448, respectively). No significant associations were found with motor severity (BFMDRS-M) or with BADS scores.

Interestingly, no significant relationships were observed between thalamic lesions and either motor or functional outcomes following GPi-DBS at 1-year follow-up, regardless of the clinical scale considered.

Correlation analyses demonstrated a significant relationship between thalamic volume and baseline functional severity. Lower thalamic volumes correlated with worse BFMDRS-F scores (right: r = −0.4142, p = 0.0023; left: r = −0.3473, p = 0.0116) and BADS scores (right: r = −0.3310, p = 0.0165; left: r = −0.3227, p = 0.0196). No correlation was found with BFMDRS-M (Supplementary Figure 3).

At 1 year post–GPi-DBS, no significant correlations were identified between thalamic volumes and improvement in motor or functional scores (BFMDRS-M, BFMDRS-F, or BADS).

SUPPLEMENTARY DISCUSSION

The retrospective design of this study should be acknowledged, as it limits strict causal inference. However, this design was appropriate given the rarity of patients undergoing GPi-DBS for dystono-dyskinetic syndromes secondary to perinatal anoxic encephalopathy, a population that remains highly selected and managed by only a limited number of specialized centers worldwide. Importantly, our cohort represents one of the largest series combining detailed volumetric MRI analyses with long-term clinical outcomes in this specific surgical indication.

Given the exploratory yet hypothesis-driven objective of identifying imaging biomarkers of DBS response, p-values were not corrected for multiple comparisons. This methodological choice was made to preserve sensitivity in analyses focused on predefined motor structures rather than on large-scale, data-driven screening. The observed correlations between putaminal volume and postoperative motor outcomes were consistent across follow-up time points, biologically plausible, and in line with established basal ganglia pathophysiology, making a purely spurious statistical effect unlikely. In contrast, thalamic volumetric measures did not show a detectable association with postoperative DBS outcomes in our cohort. Thalamic analyses were therefore considered exploratory and are reports as complementary findings.

Regarding the post-hoc analyses, the focus on the identification of clinical markers associated with severe putaminal atrophy, with only indirect relevance to DBS outcome.

The absence of measurable postoperative improvement in a subset of patients (n=14) including some without putaminal atrophy, likely reflects a combination of heterogeneous factors, including mixed dystonic-pyramidal features in some cases (e.g.subtle or subclinical spasticity, which was managed with oral baclofen when clinically indicated), fixed musculoskeletal adaptations, and limitations of current dystonia rating scales in severe or complex presentations.

Beyond these statistical considerations, the evolving diagnostic landscape also warrants attention. This evolution reinforces the concept that “cerebral palsy” represents a heterogeneous clinical label rather than a unified etiological entity. In the present cohort, extensive MRI evidence of perinatal hypoxic-ischemic injury, including basal ganglia involvement and putaminal atrophy, supported the diagnosis of secondary DDS related to perinatal anoxia rather thant a primary or genetic dystonia.

At the same time, genetic testing strategies have evolved substantially over the study period, and the increasing recognition of genetics mimics of cerebral palsy highlights the importance of comprehensive etiological assessment, including genetic testing when appropriate, in contemporary DBS candidate selection. More broadly, this evolution supports a shift toward mechanism-based stratification of patients considered for neuromodulation.

Nevertheless, these findings should be interpreted as evidence of a strong and clinically meaningful association rather than definitive proof of causality. Future prospective and multicentric studies will be useful to confirm these results and refine statistical correction strategies. Overall, our data support putaminal integrity as a robust imaging predictor of GPi-DBS efficacy in DDS secondary to PAE.

Our findings suggest that the extent of putaminal atrophy significantly undermines DBS efficacy by disrupting these key neurochemical pathways. The putamen plays a critical role in motor control, particularly within the basal ganglia-thalamo-cortical loops. It integrates motor signals from the cortex to facilitate smooth, coordinated movements. In dystono-dyskinetic syndromes, putaminal atrophy disrupts these circuits, contributing to motor dysfunction(1). Neurochemically, the putamen relies on key neurotransmitters like GABA, which regulates basal ganglia activity. GABAergic projections from the putamen to the GPi are crucial for controlling GPi excitatory output to the thalamus and cortex. In putaminal atrophy, the loss of GABAergic neurons reduces GPi inhibition, leading to dysregulated motor activity typical of dystonia and dyskinesia(2). Dopamine also plays a significant role in modulating the activity of the putamen, particularly through D1 and D2 dopamine receptors, which are differentially expressed in the direct and indirect pathways of the basal ganglia(3–6). These pathways fine-tune motor commands, with D1 receptors facilitating movement through the direct pathway (GPi), and D2 receptors inhibiting it through the indirect pathway (GPe/STN/GPi). Damage to the putamen can disrupt the balance between these pathways, contributing to the abnormal motor control seen in dystono-dyskinetic syndromes. This disruption may also explain why DBS, which modulates abnormal GPi activity, is less effective in patients with extensive putaminal damage(7). The predominance of excitatory receptors in the motor putamen can explain its specific sensitivity to anoxic aggression (excito-toxicity) at birth while GPi remains much less concerned by this initial process due to its GABAergic constitution.

Putaminal volumetry likely reflects not only lesion burden but also the integrity of critical afferent node to the motor thalamus, which has been identified as the dominant connectivity hub in lesion-derived network of dyskinetic cerebral palsy(8).

Volumetric assessment of deep brain structures remains a methodological challenge. Segmentation can be performed manually, semi-automatically, or using deep learning, each approach having specific advantages and limitations. Kim et al. (2024) showed that automated deep learning segmentation (VUNO Med-DeepBrain) is fast and reproducible but tends to underestimates subcortical volumes—particularly in young children—compared with FreeSurfer combined with manual correction(9).

Because these regions are central to HIE and DBS outcome prediction, we tailored our methods by structure: manual Horos volumetry for the putamen (allowing exclusion of atrophied areas), Brainlab for thalamic mapping with manual verification, and CAT12 for cortical gray matter. CAT12/SPM pipelines have been validated in pediatric populations, when age-specific templates are used. Although normalization errors are more frequent in children under 6 years(10), all patients in our cohort were older, minimizing this limitation. In this age range, CAT12 has shown robust accuracy and good agreement with SPM12(11–13).

Normalization to total gray matter, rather than TIV, provided a more physiologically meaningful correction in children and minimized bias, as previously described by O’Brien et al.(14). These methodological refinements enhance the reliability of our volumetric analyses and reinforce the validity of putaminal atrophy as a biomarker of DBS outcome prediction.

REFERENCES

1. Monbaliu E, Himmelmann K, Lin JP, Ortibus E, Bonouvrié L, Feys H, et al. Clinical presentation and management of dyskinetic cerebral palsy. Lancet Neurol. 2017 Sep;16(9):741–9.

2. Calabresi P, Picconi B, Tozzi A, Ghiglieri V, Di Filippo M. Direct and indirect pathways of basal ganglia: a critical reappraisal. Nat Neurosci. 2014 Aug;17(8):1022–30.

3. Alexander GE, Crutcher MD. Functional architecture of basal ganglia circuits: neural substrates of parallel processing. Trends Neurosci. 1990 Jul;13(7):266–71.

4. DeLong MR, Wichmann T. Basal Ganglia Circuits as Targets for Neuromodulation in Parkinson Disease. JAMA Neurol. 2015 Nov;72(11):1354–60.

5. Wichmann T, DeLong MR. Deep Brain Stimulation for Movement Disorders of Basal Ganglia Origin: Restoring Function or Functionality? Neurotherapeutics. 2016 Apr;13(2):264–83.

6. Gerfen CR, Surmeier DJ. Modulation of striatal projection systems by dopamine. Annu Rev Neurosci. 2011;34:441–66.

7. Kupsch A, Tagliati M, Vidailhet M, Aziz T, Krack P, Moro E, et al. Early postoperative management of DBS in dystonia: programming, response to stimulation, adverse events, medication changes, evaluations, and troubleshooting. Mov Disord. 2011 Jun;26 Suppl 1:S37-53.

8. de Almeida Marcelino AL, Al-Fatly B, Tuncer MS, Krägeloh-Mann I, Koy A, Kühn AA. Lesion distribution and network mapping in dyskinetic cerebral palsy. Brain Commun. 2025;7(3):fcaf228.

9. Kim MJ, Hong E, Yum MS, Lee YJ, Kim J, Ko TS. Deep learning-based, fully automated, pediatric brain segmentation. Sci Rep. 2024 Feb 22;14(1):4344.

10. Muzik O, Chugani DC, Juhász C, Shen C, Chugani HT. Statistical parametric mapping: assessment of application in children. Neuroimage. 2000 Nov;12(5):538–49.

11. Sargolzaei S, Sargolzaei A, Cabrerizo M, Chen G, Goryawala M, Pinzon-Ardila A, et al. Estimating Intracranial Volume in Brain Research: An Evaluation of Methods. Neuroinformatics. 2015 Oct;13(4):427–41.

12. Farokhian F, Beheshti I, Sone D, Matsuda H. Comparing CAT12 and VBM8 for Detecting Brain Morphological Abnormalities in Temporal Lobe Epilepsy. Front Neurol. 2017;8:428.

13. Lee SM, Kim E, You SK, Cho HH, Hwang MJ, Hahm MH, et al. Clinical adaptation of synthetic MRI-based whole brain volume segmentation in children at 3 T: comparison with modified SPM segmentation methods. Neuroradiology. 2022 Feb;64(2):381–92.

14. O’Brien LM, Ziegler DA, Deutsch CK, Frazier JA, Herbert MR, Locascio JJ. Statistical adjustments for brain size in volumetric neuroimaging studies: some practical implications in methods. Psychiatry Res. 2011 Aug 30;193(2):113–22.
